# Facts and Figures

**Published:** 1888-06-02

**Authors:** 


### FACTS AND FIGURES.

The narrative which we publish this week in place of our usual series of articles and news is entirely a narrative of facts; the tables at the end furnish a record of figures. This simple narrative and those tables of figures taken together, afford probably a better index to the condition of the moral and social condition of the people of London than any other similar quantity of printed matter which can be found anywhere. The tables show the number and necessities of the sick poor, and the vast amount of money spent annually in their relief; the narrative describes very fully how it is all done. Together they furnish, as in a picture, a birdseye view of the whole field of metropolitan hospital operations and of the workers therein.

It may be taken for granted that a large number of people who are not at all familiar with hospitals and their work will be awakened to a little temporary attention to the subject on Hospital Sunday. If they could be induced to make such a study of the work as it really deserves we do not know where they would find in briefer form or in fuller detail the kind of information they require for their purpose so easily as in this carefully-compiled narrative and those carefully-constructed tables. We have no means of knowing how far the facts and figures here set forth may be of interest to the general public. But that they are compendious and reliable we are very certain. We think we may ask preachers and speakers, without any straining of modesty, to strive to stir up an awakened interest in hospitals on the lines here laid down. The facts are of the most deeply-interesting character, and the figures are the crown and completion of the facts. London may be justly proud of both. The striking thing about them is that they are a kind of systematised result of a totally unsystematised series of operations. The work of the London hospitals is almost exclusively the work of free lances and volunteers working separately. Nothing shows better the line of duty and the inherent sense of practical capacity which characterise Englishmen than our hospital system. Without any general plan order is evoked; without any prearranged purpose efficiency is created. The metropolitan hospital system is no system at all, and yet it produces results of the highest excellence, and of the most surprising magnitude.

But the system, or no system, is becoming unwieldy in its vastness; and its want of compactness and uniformity is likely soon to diminish its general efficiency. The public must now take it in hand. A few men have laboured to establish it; the whole population must enter into their labours. What a glorious legacy the hospital workers of past times have left for the men of the present generation! How nobly we are exalted above the ancient civilisations of Greece and Rome by the possession of our hospital system! What a proud pre-eminence it gives us even over our Continental neighbours who are contemporaneous with us! In no country—not even in the English-speaking continent of America—has the voluntary system of medical charity reached such developments as with us. Is it possible to imagine that the zenith has been reached, and that decay is approaching? It is possible to believe this, if we can believe that England has passed her meridian, and is now only a setting sun. It is possible to imagine it, if we can believe that our capacity for faith in a noble creed and our power to embody the principles of that creed in fit works of benevolence and duty is exhausted. But if Englishmen still are—what they always have been—men of earnest conviction, resolute purpose, and an intelligent love of duty, we feel firmly assured that now as ever, in the future as in the past, the poor will be helped, the sick will be cared for, the suffering will be relieved. If England declines, hospitals may decline with her; but so long as she carries her head erect and advances with firm tread, so long will hospitals share her growing strength and her increasing prosperity.

